# Late Cardiac Tamponade in a Patient Victim of Penetrating Trauma – Case Report

**DOI:** 10.21470/1678-9741-2019-0130

**Published:** 2020

**Authors:** Adnaldo da Silveira Maia, Alexandre Bichara da Cunha, Magnum Adriel Santos Pereira, Natalia Pompeu Chaves, Ricardo Silva de Morais, Lázaro Araújo de Almeida

**Affiliations:** 1 University of Amazonas State, Manaus, AM, Brazil.; 2 Adriano Jorge Hospital Foundation, Manaus, AM, Brazil.; 3 Nilton Lins University, Manaus, AM, Brazil.; 4 Dr. João Lúcio Pereira Machado Emergency Hospital, Manaus, AM, Brazil.

**Keywords:** Cardiac Tamponade, Stab Wounds, Pericardial Window Techniques

## Abstract

Case Presentation: A case of a 49-year-old patient, male, victim of stab wound, developing belatedly cardiac tamponade and hemodynamic stability was reported. The patient underwent a pericardial window with drainage of pericardial effusion of blackened aspect; however, without visualization of the cardiac lesion, enlargement of the incision by median sternotomy was opted for. A hematoma was spotted at the left ventricle with epicardial lesion and a patch of pericardium was made with 3-0 polypropylene. The patient developed acute pulmonary edema and atrial fibrillation, which improved after the intensive care unit clinical management, with hospital discharge in the 7^th^ postoperative day.

**Table:** 

Abbreviations, acronyms & symbols	
ATLS	= Advanced Trauma Life Support
FAST	= Focused Assessment with Sonography in Trauma
ICU	= Intensive care unit
NIV	= Noninvasive ventilation

## INTRODUCTION

Cardiac trauma is a challenge for surgeons. Penetrating lesions are responsible for high mortality ranging from 16 to 97%. Major causes of death include cardiac tamponade and hypovolemic shock, which require identification and immediate action from the trauma team^[1]^.

Numerous patients come to the emergency department with penetrating or blunt trauma. Many die during transport or at the trauma scene. Cardiac tamponade is known to have a protective effect, restricting massive volume loss, saving time for transportation and surgical approach. The surgeon needs a high index of suspicion in those cases with hemodynamic stability^[2]^.

Because available diagnostic methods and initial management when cardiac trauma are suspected, late cardiac tamponade is rare and may occur up to 100 days after trauma and can have an atypical presentation. The etiology of delayed cardiac tamponade is unclear, although some mechanisms have been proposed to explain, including clot sealing a partial tear that allows a leak into the pericardial sac^[3]^.

The injuries that most cause the possibility of cardiac trauma are located in the anterior chest wall, specifically in the Ziedler area, particularly victims of penetrating trauma with transfixing chest injuries^[2]^. Beck’s triad represents the main clinical feature suggesting the diagnosis, however, identified in up to 33% of patients^[4,5]^. The most common chamber involved is the right ventricle due to its anatomical location^[6]^.

The pericardial window is an alternative for the diagnosis of penetrating cardiac injury in hemodynamically stable patients^[7]^.

We present a case of perforating trauma in the Ziedler area, evolving 52 days later with important cardiac tamponade and hemodynamic stability.

## CASE REPORT

A 49-year-old male patient presented at the emergency department of a small city in the Brazilian Amazon with a penetrating trauma in the left inframammary region (7^th^ intercostal space) in December 2018. He underwent a closed chest drainage in his city, remaining hospitalized for 5 days with progressive recovery and eventual hospital discharge.

Two months later, the patient returned to the emergency department with dyspnea, dry cough, fever and syncope. At this occasion, he was diagnosed with pneumonia and treated with antibiotics, being discharged after 4 days.

Eight days later, he once again returned to the emergency department with a significant pericardial effusion. The team, without success, made several attempts of pericardiocentesis. The patient was transferred to the emergency service in Manaus.

**Fig. 1:**
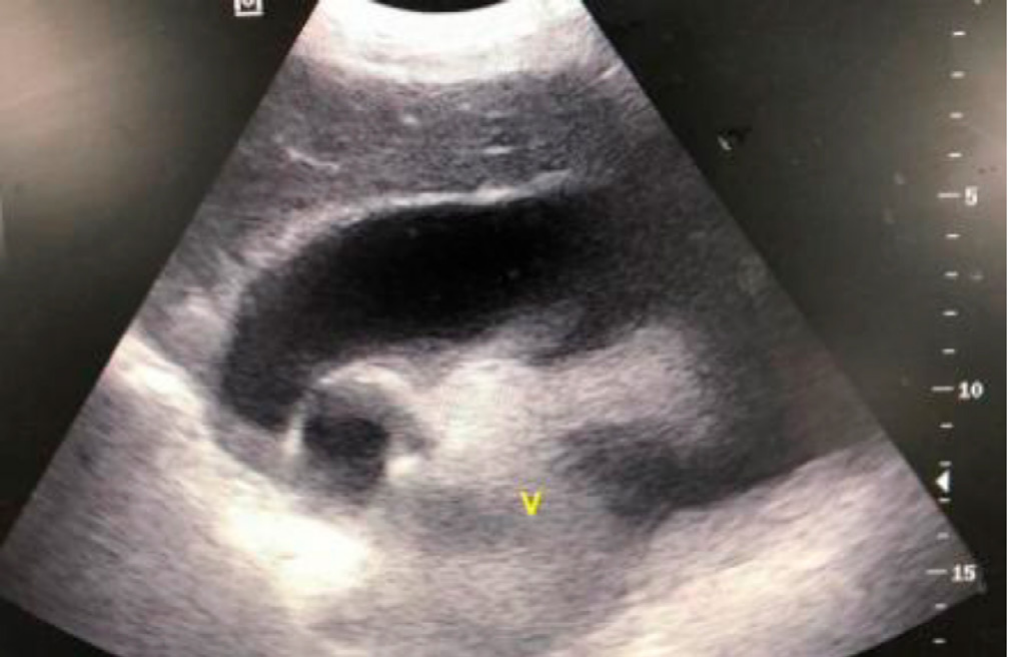
Pericardial effusion visualized in the trauma center during FAST.

Upon arrival, he was conscious, oriented, eupneic, hemodynamically stable, acyanotic, presenting capillary filling time of less than 2 seconds, bilateral jugular turgor, pulse rate 84 bpm and blood pressure of 137/94 mmHg. On auscultation, he presented a reduced vesicular murmur in the left pulmonary base and muffled heart sounds. Focused assessment with sonography in trauma (FAST) showed pericardial effusion (Figure 1). Admission laboratory results showed hemoglobin of 8.7 g/dL and hematocrit of 27.5%.

The patient was submitted to a pericardial window with a subxiphoid median incision of approximately 10 cm. Aspiration of 1500 mL of dark red blood was performed without the possibility of visualization of the cardiac lesion. The incision was enlarged from the sternotomy to the manubrium using a bone cutter.

**Fig. 2:**
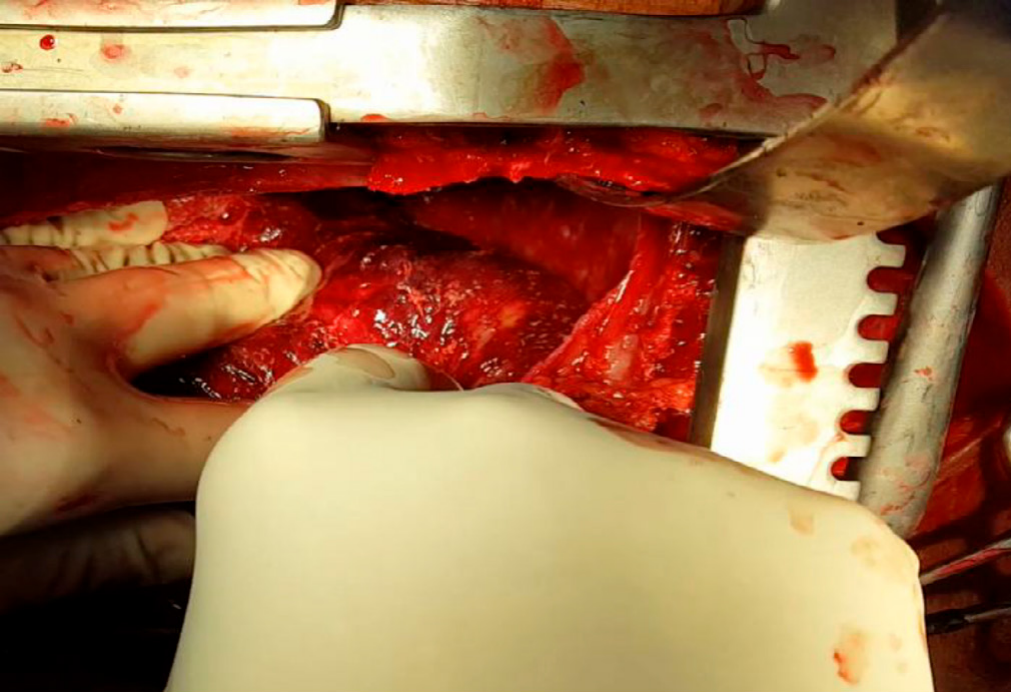
Hematoma in the posterior region of the left ventricle associated with epicardial lesion.

**Fig. 3:**
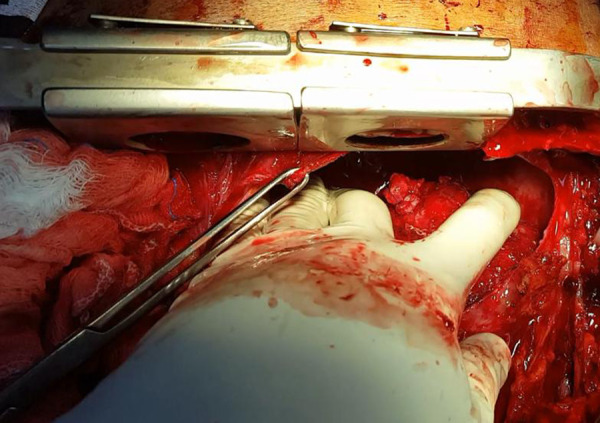
Final appearance after pericardial patch on the topography of lesion with 3-0 polypropylene suture.

A large amount of fibrin was seen on the epicardial surface and hematoma in the posterior region of the left ventricle with epicardial lesion (Figure 2). The surgical team (Figure 3) performed a patch on the topography of the lesion with 3-0 polypropylene suture and epicardial closure with 3-0 polyglactin 910 suture in layers. A 24-Fr chest tube was inserted into the pericardial cavity.

The patient was referred to the intensive care unit (ICU), evolving on the 1^st^ postoperative day with acute pulmonary edema and high-throughput atrial fibrillation requiring diuretics, vasodilators, amiodarone and noninvasive ventilation (NIV), with significant improvement. He was discharged from the ICU on the 3^rd^ postoperative day and was discharged from hospital on the 7^th^ day without recurrence.

## DISCUSSION

Penetrating heart injuries have been described for centuries. In Brazil, Sylvio Brauner performed the first cardiac wound repair in 1927, in an emergency room in Rio de Janeiro. In 1942, Euryclides de Jesus Zerbini performed cardiorrhaphy in a patient with anterior descending artery occlusion caused by metallic splinter^[1,5]^.

The trauma resulting from stab wounds has a higher frequency and is more likely to result in a better outcome when compared to firearm-related injuries. In this type of trauma, 80% of the patients are affected in the Ziedler area. Regarding the anatomical site of lesion, right ventricle is the most affected chamber (43%), followed by left ventricle (34%) and right atrium (18%)^[1]^. Our case demonstrates left ventricular injury despite its high intracavitary pressure and hemodynamic status of the patient.

Cardiac tamponade is often associated with stab injury, clinically evidenced by the Beck’s triad, which is present in a minority of patients (low blood pressure, muffled heart sounds and increased jugular venous pressure)^[3,4]^. In the present case, despite the last two criteria present, the patient's blood pressure was slightly elevated.

The approach of these patients is initiated by the Advanced Trauma Life Support (ATLS), with clinical stabilization. Pericardial window is considered by many authors as an option in the diagnosis of cardiac injuries, presenting high sensitivity and specificity as well as convenience during the operative event, allowing the surgeon to enlarge or close the incision^[5,7]^.

In a small number of cases, the patient is hemodynamically stable with signs of cardiac tamponade resulting from a late bleeding with an already healed pericardium after the acute injury or by the development of pericarditis, resulting in challenge for the surgical team^[2,5]^. This condition was evidenced in this report.

Thus, we also highlight the delayed diagnosis of the patient, previously treated with antibiotic therapy and diagnostic hypothesis of pneumonia, as well as the distances and shortage of diagnostic resources faced by the state population.

**Table:** 

Author's roles & responsibilities	
ASM	Acquisition, analysis, or interpretation of data for the work; drafting the work or revising it critically for important intellectual content; final approval of the version to be published
ABC	Substantial contributions to the conception or design of the work; drafting the work or revising it critically for important intellectual content; final approval of the version to be published
MASP	Substantial contributions to the conception or design of the work; drafting the work or revising it critically for important intellectual content; final approval of the version to be published
NPC	Acquisition, analysis, or interpretation of data for the work; drafting the work or revising it critically for important intellectual content; final approval of the version to be published
RSM	Substantial contributions to the conception or design of the work; drafting the work or revising it critically for important intellectual content; final approval of the version to be published
LAA	Substantial contributions to the conception or design of the work; drafting the work or revising it critically for important intellectual content; final approval of the version to be published
